# Case report: Elective management of a bicornuate uterus with hematometra, incidentally found submucosal fibroids, rectus sheath hematoma, and hydronephrosis in a resource limited setting

**DOI:** 10.1016/j.ijscr.2024.109889

**Published:** 2024-06-07

**Authors:** Anusha Ashkar, Saba Shah, Fizza Yousuf, Fatima Zulfiqar Siddiqui, Hamza Yousuf

**Affiliations:** Dow University of Health Sciences, Karachi, Pakistan

**Keywords:** Bicornuate, Uterus, Hematometra, Hematoma, Fibroids, Mullerian

## Abstract

**Introduction and importance:**

Bicornuate uterus is a rare type of congenital mullerian anomaly, presenting as a diagnostic challenge. Metroplasty either via an open approach or laparoscopically can be performed to definitively diagnose and treat the defect.

**Case presentation:**

A 26-year-old female, with no known comorbids and past surgical history of endometriotic cyst excision presented with acute symptoms of left lower abdominal pain, burning micturition, and relative constipation. After clinical and radiologic investigations, the diagnosis of bicornuate was suspected. An elective exploratory laparotomy was then performed due to limited resources and skilled surgical techniques. Intra-operatively it was found that she had a bicornuate uterus with a single cervix and vagina, with the left cornuate being non-communicating with fluid suggesting hematometra. Dense adhesions were reported with drainage of 150–200 ml of free fluid upon opening the rectus sheath. Postoperatively she remained vitally stable and was discharged home.

**Clinical discussion:**

We report a rare case of a bicornuate uterus with double horns along with submucosal fibroids, rectus sheath hematoma, and left sided hydronephrosis. Diagnosis of bicornuate uterus is associated with diagnostic uncertainty mainly due to its rarity and nonspecific presentation.

**Conclusion:**

Although bicornuate is rare, it may result in complications if not attended to timely. Early diagnosis and management are necessary to minimize associated morbidity and mortality that can occur as a consequence of associated unattended pressure symptoms.

## Highlights

• Bicornuate uterus is a rarely report mullerian duct anomaly.
• The diagnosis and therapeutic management present as a challenge especially in a resource limited setting.
• The patient underwent exploratory laparotomy with total abdominal hysterectomy as the end outcome.
• The patient was fully aware of the consequences upon fertility and gave her informed consent.

## 1 Introduction

Congenital alterations in the mullerian anatomy are reported to be 0.5 % to 6.7 % in the general population with incidence rising to 16.7 % in women with miscarriages [1]. Bicornuate uterus, a subtype (according to the classification, a mullerian anomaly type IV), is defined as an incomplete fusion of Mullerian ducts leading to the development of a heart-shaped uterus instead of a pear shape [2]. Etiology remains multifactorial, including both genetic factors such as mutations of a subset of homeobox genes along with Wnt7a, necessary for differentiation of mullerian ducts, and environmental factors [3].

The women with bicornuate uterus may remain asymptomatic or present with vague symptoms of menorrhagia, pelvic pain, and dysmenorrhea. Renal anomalies may coexist with the Mullerian anomalies with superimposed renal symptoms, however, a bicornuate uterus with non-communicating uterine cavities may be linked with renal agenesis, resulting in pronounced pressure symptoms like urinary retention and excruciating pelvic pain [[4], [5], [6]].

The rarity of bicornuate uterus presents as a diagnostic and therapeutic challenge. This case report presents a case of bicornuate uterus with hematometra, rectal sheath hematoma with hydronephrosis and other pressure symptoms, treated via an explorative laparotomy approach in an elective setting. This case reports conforms with the SCARE guidelines [7].

## 2 Case presentation

A 26-year-old female, divorced, nulliparous with no known comorbidities, with past surgical history of removal of an endometriotic cyst two years back, presented to our teaching hospital with recurrent symptoms of intermittent lower abdominal pain and constipation which were managed conservatively over the past six months. However, her current presentation was marked by acute symptoms, including:

1) Lower left sided abdominal pain radiating towards the left flank region for six days.
2) Burning micturition for four days.
3) Relative constipation with passage of flatus for four days.

The patient has had a non-significant gynecological history with a 6/28-day menstrual cycle. Upon admission, her vital signs were recorded as follows: GCS of 15/15, a pulse of 95/min, blood pressure of 100/80, non-pyrexial, respiratory rate of 22/min, and maintained oxygen saturation on room environment. Physical abdominal examination revealed a distended abdomen, moderate tenderness in the left flank region, and a fixed mass arising from the pelvis not separately felt from the uterus.

Laboratory data demonstrated normal full blood count, urine detailed report, renal functions, and electrolytes.

**Supplementary Fig. 1:**
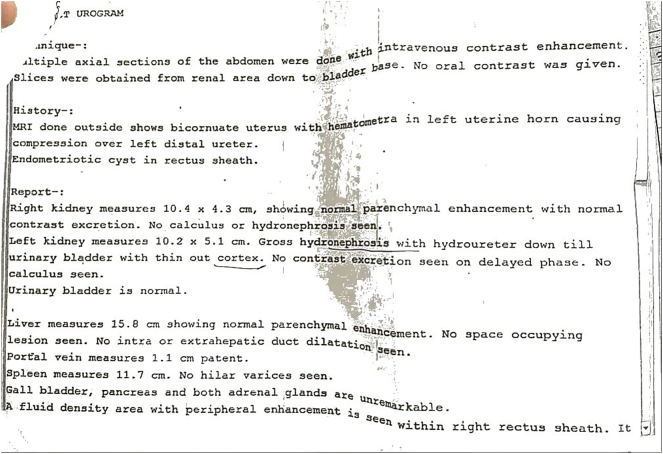
Radiological imaging report.

**Supplementary Fig. 2:**
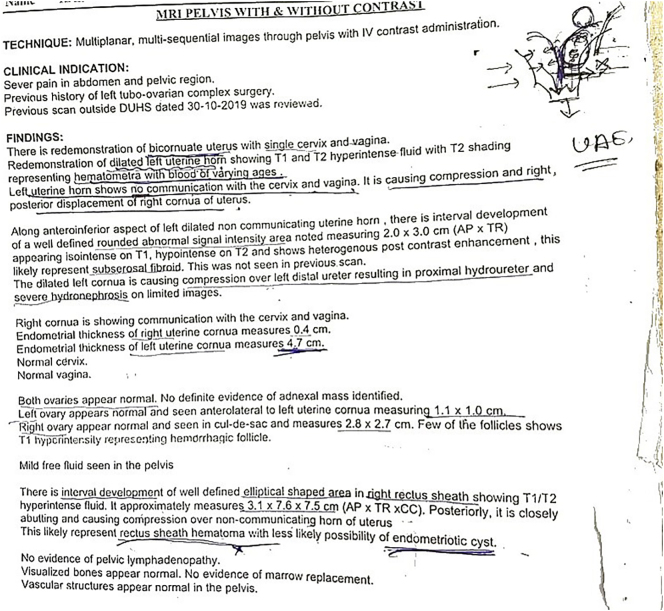
MRI pelvis with/without contrast.

Initial KUB ultrasound imaging disclosed left sided hydronephrosis with loss of corticomedullary distinction. Subsequently a CT urogram was performed, suggesting a left sided hydronephrosis with a hydroureter down to urinary bladder without contrast excretion. Furthermore, a bicornuate uterus with hematometra in left horn and an endometriotic cyst along the right rectus sheath was revealed. An MRI was performed later, which conformed with CT urogram findings, reporting a bicornuate uterus with a single cervix and vagina, with the left cornuate being non-communicating, having T1 and T2 hyperdense fluid suggesting hematometra; a rectus sheath hematoma was also demonstrated, compressing the left cornuate uterus. There was mild free fluid in the abdominopelvic cavity. Diagnosis of a bicornuate uterus with left rectus sheath hematoma and pressure symptoms resulting in hydronephrosis was made and to address the patient's condition, patient received intravenous (IV) fluid resuscitation and pain was managed accordingly (Supplementary Fig. 1, Supplementary Fig. 2).

After explaining the patient's diagnosis to her and the necessity for surgery, she provided written and informed consent.

**Fig. 1:**
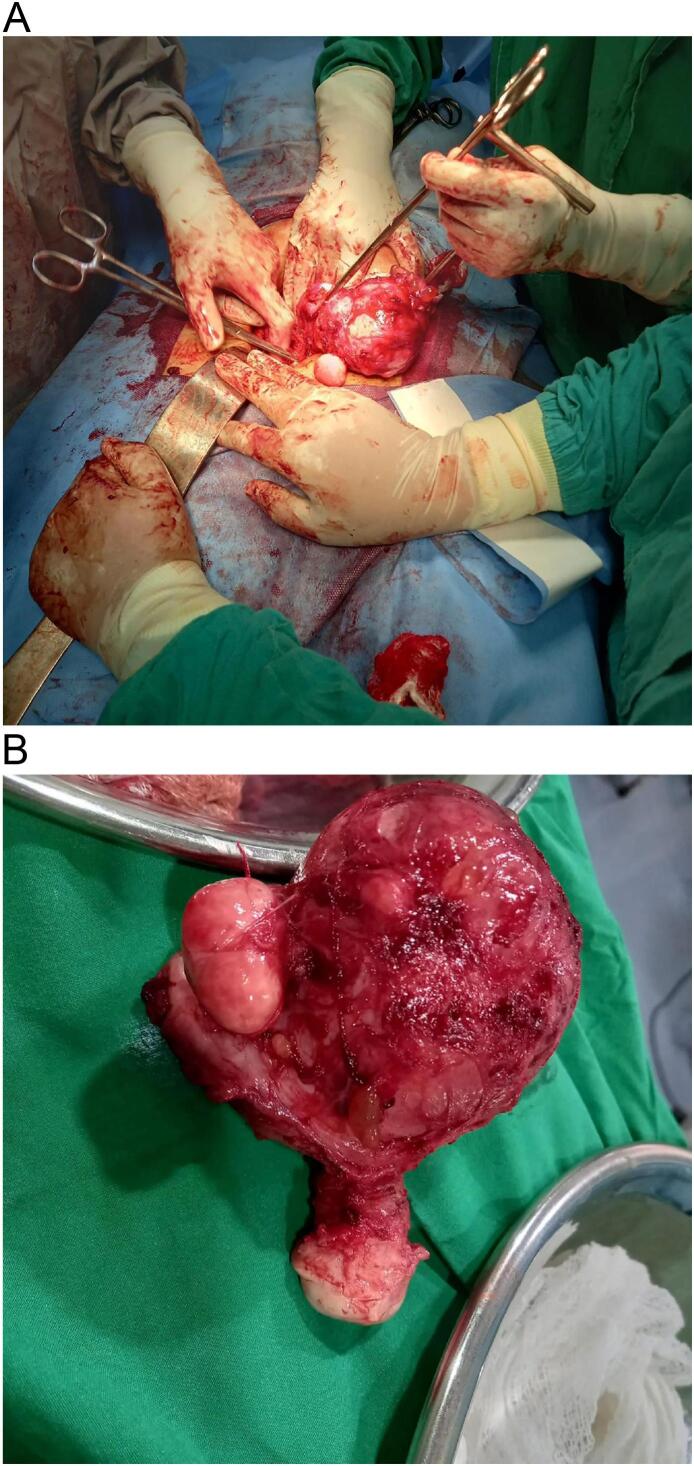
A: Intraoperative image of exploratory laparotomy. B: Bicornuate uterus successfully excised.

An exploratory laparotomy was performed with total abdominal hysterectomy (TAH), right sided ovarian cystectomy and salpingectomy, and repair of gut serosal tear. Dense adhesions were reported in the pelvic anatomy with drainage of 150–200 ml chocolate colored fluid upon opening the rectus sheath. Two subserosal fibroids, the largest measuring 2.5 cm was found in the right cornuate uterus. During adhesiolysis, a serosal tear of the gut measuring 2 × 2.5 cm also was repaired (Fig. 1A and B).

Following the successful surgical intervention, the patient remained vitally stable and was discharged home with follow-ups in OPD (Fig. 1A and B).

## 3 Discussion

Among the congenital uterine structural anomalies reported, bicornuate uterus, a unification defect is rarely reported with a prevalence of just 0.4 % in the general population with statistics slightly increasing for women with infertility and subsequent miscarriages [8]. Even rare is the reporting of bicornuate uterus in South East Asia, thus we present this case of bicornuate uterus with hematometra, rectal sheath hematoma, and superimposed hydronephrosis due to pressure symptoms, the first of its kind case in Pakistan.

Initially classified by Buttram and Gibbons and later revised by the ‘American Society of Reproductive Medicine’ in late 1988, Müllerian anomalies are classified into seven classes, of which Class IV is bicornuate (IV a is complete and IV b is partial) [2]. However, the rarity of its reporting leads to uncertainty regarding its diagnostic certainty and therapeutic management.

The three widely accepted imaging modalities includes i) Ultrasound, the most common imaging modality performed at the first visit; ii) Hysterosalpingography, a contrast-based imaging usually utilized in cases of subfertility; and iii) MRI, the best modality merely because of its noninvasiveness, production of images in different planes, absence of radiation, and ability to rule out fusion anomalies from resorption anomalies, thus narrowing down diagnostic discrepancies [9,10]. It is reported that a split of 10 mm or more of the uterine fundus on MRI is highly suggestive of a fusion anomaly, precisely, a bicornuate uterus [10].

While the management of a bicornuate uterus is mainly managed by either an open approach i.e. Strassmann metroplasty or a laparoscopic approach, which is preferable to Strassman due to decreased reporting of intra-operative blood loss, duration of surgery, risk of infections, and postoperative adhesions formation; however, in case of pregnancy, the aggressive antenatal monitoring is recommended to mitigate obstetric complications [11,12]. In our case, an exploratory laparotomy with subsequent TAH was performed because of the emergent nature of the case, lack of expertise, and necessary resources. It must be made known that the patient was fully aware of the reproductive outcomes of TAH and gave her full consent.

While the complications of bicornuate uterus can be varied during pregnancy, ranging from preterm labor to postpartum hemorrhage, the risk of uterine rupture remains inevitable, mainly reasoned because of the presence of a fibrous band that restraints the uterus from expanding hence making it prone to rupture [13].

It must be emphasized that due to a unification defect, a biopsy sample, if taken from the healthy endometrium may report a false-negative result, leading to a poor prognosis [14]. Thus, care must be taken, and radiological imaging guidance must be utilized to prevent such occurrences.

While mullerian anomalies may occur independently, available literature draws a link between endometriosis and the occurrence of mullerian defects supporting the coelomic and Sampson's retrograde menstruation theory among others [15]. The occurrence of bicornuate uterus in our case report draws parallels with the previously published literature showing the possible cause-effect model between endometriosis and mullerian defects [[16], [17], [18], [19]].

It is postulated that mullerian anomalies are also associated with renal anomalies however in our case the concurrent occurrence of hydronephrosis and hydroureters leads to malfunctioning of the renal system resulting in recurrent urinary tract infections (UTIs), leading to structural and defects of physiology.

Much emphasis is laid on the occurrence of bicornuate uterus and the associated pathological sequelae; however, extensive literature also supports the hypothesis that hydronephrosis and hydroureters may result from endometriotic dense deposits [20,21]. According to Nezhat et al. in a case series, all three cases reported compromised renal function with hydronephrosis and hydroureters secondary to endometriosis that led to procedures like laparotomy and laparoscopy to salvage the renal function [22]. In a retrospective cohort of 436 patients undergoing laparoscopic deep endometriosis (DE) surgery, statistically significant difference was observed between renal loss group and control in terms of ureteral DE (*p* < 0.0001) [23]. Since our laparotomy also revealed dense adhesions, endometriosis could be linked to the occurrence of compromised renal function and the associated pressure symptoms of the urinary tract.

## 4 Limitations

1) The rectus sheath hematoma was diagnosed in a stepladder fashion with the usage of ultrasound study followed by MRI pelvis with contrast, as the radiological imaging. The medical team was confident with the diagnosis of rectus sheath hematoma as a biopsy/needle aspiration even if arranged, could have risked in the puncture of the bladder or bowel [24,25]. The drainage of 150–200 ml of fluid upon opening the rectus sheath during laparotomy also supports the diagnosis of rectus sheath hematoma.
2) Previous hospital admissions/surgical procedures related documents could not be retrieved hence little remains known about the details of the removal of endometriotic cyst procedure, performed two years back.

## 5 Conclusion

Our case report reflects upon the rarely reported bicornuate uterus and the therapeutic management in our resource limited setting, contrasting with the set guidelines.

The following are the supplementary data related to this article.

## Consent

Written informed consent was obtained from the patient for publication and any accompanying images. A copy of the written consent is available for review by the Editor-in-Chief of this journal on request.

## Ethical approval

This case report is observational in nature henceforth given exemption by the ethics review committee of ‘Dow University of Health Sciences’.

## Funding

No funding acquisition.

## Author contribution

Anusha Ashkar (1); conceptualisation; drafting original and review/editing, methodology.

Saba Shah (1); data curation, supervision.

Fizza Yousuf (1); project administration.

Fatima Zulfiqar Siddiqui (1); manuscript writing/drafting/editing.

Hamza Yousuf (1) resources.

## Guarantor

Dr. Anusha Ashkar and Dr. Saba Shah.

## Conflict of interest statement

None.
